# Physicochemical Characterization and Dissolution Properties of Meloxicam-Gelucire 50/13 Binary Systems

**DOI:** 10.3797/scipharm.1101-22

**Published:** 2011-04-10

**Authors:** Mahmoud El-Badry

**Affiliations:** 1 Department of Pharmaceutics, College of Pharmacy, King Saud University, P.O. BOX 2457, Riyadh 11451, Saudi Arabia; 2 Department of Pharmaceutics, Faculty of Pharmacy, Assiut University 71526, Assiut, Egypt

**Keywords:** Solid dispersion, Meloxicam, Spray drying, Dissolution, Anti-inflammatory activity, Powder X-ray diffractometry analysis, Scanning electron microscopy

## Abstract

A solid dispersion of Meloxicam (MX), a poorly soluble, non steroidal anti-inflammatory drug, and Gelucire 50/13 was prepared by spray drying. Spherical microparticles were yielded with smooth surfaces as observed by scanning electron microscopy. According to differential scanning calorimetry and powder X-ray diffractometry analysis, MX was transformed from the crystalline state to the amorphous state as confirmed by the disappearance of its melting peak and the crystalline peaks. The dissolution tests at pH 7.4 revealed that the dissolution rate of encapsulated MX was 2.5-fold higher than that of the corresponding physical mixture and fourfold higher than the drug alone, respectively. The microparticles prepared at a ratio of 1:4 (drug/Gelucire) exhibited a 4-fold higher anti-inflammatory activity on the paw edema of rats in comparison to the drug alone. All in all, this work reveals that spray drying is a suitable technique for preparation of solid dispersions with improved biopharmaceutical and pharmacological characteristics of MX.

## Introduction

Meloxicam (MX), 4-hydroxy-2-methyl-*N*-(5-methyl-1,3-thiazol-2-yl)-2*H*-1,2-benzothiazine-3-carboxamide 1,1-dioxide, is a non-steroidal anti-inflammatory drug (NSAID). MX is a potent inhibitor of cyclogenase (COX), and in several models exhibits selectivity for the inducible isoenzyme COX2. It is used to treat rheumatoid arthritis, osteoarthritis and ankylosing spondylitis. [1–4]. Similar to many other NSAIDs, MX is practically insoluble in water and can be graded in class II, of the Biopharmaceutical Classification System, which means low aqueous solubility and rapid absorption (high permeability) through gastrointestinal tract [5]. Gelucire 50/13 is an excipient composed of fatty acid (C16 and C18) esters of glycerol, PEG esters and free PEG. It melts at approximately 50°C and has hydrophilic-lipophilic balance (HLB) value of 13 [6, 7]. Gelucires found broad application in pharmaceutical formulations e.g. the preparation of fast release and sustained release formulations [6]. Gelucires are among the several carriers having been employed in preparing solid dispersions [8–10].

The spray-drying technique is extensively used in the pharmaceutical industry to produce raw drug, excipients or microparticles, as an alternative to emulsification methods. This technique transforms the liquid feed into a dry powder in one step and is feasible for the scaling-up of the microencapsulation, continuous particle processing operations and can be applied to a wide variety of materials. Spray drying can be also used to enhance the solubility and improve particulate design [11–14].

The purpose of the current study is to characterize the solid-state properties of the solid dispersion system of Meloxicam in Gelucire 50/13 prepared at different ratios using the spray drying technique. The methods for characterization were scanning electron microscopy (SEM), differential scanning calorimetry (DSC), and powder X-ray diffraction (XRD). Moreover, solubility and dissolution studies were performed to compare the characteristics of the solid dispersion with the pure drug or physical mixture (PM). Also, the anti-inflammatory activity of the microparticles on the hind paw edema of rats was studied.

## Results and discussion

Spray drying is a rapid and simple process to prepare microparticles. The setting of different parameters, however, can affect the quality of final product. In addition, the rapid evaporation of the solvent from the droplets will influence the crystalline structures of spray dried glycerides [15]. To overcome the melting of the produced microparticles on the surface of the collector vessel of the spray drying apparatus during the process was attempted to be overcome by addition of Aerosil 200. Different drug/carrier ratios were used in this study. Spray drying at the parameters set yielded microparticulate solid dispersions (SD) with 70–75 % yield and the entrapment efficiency of the drug amounted to 90 –95 %. Therefore, the spray drying method is applicable for the preparation of SD.

### Scanning electron microscopy (SEM)

**Fig. 1.**
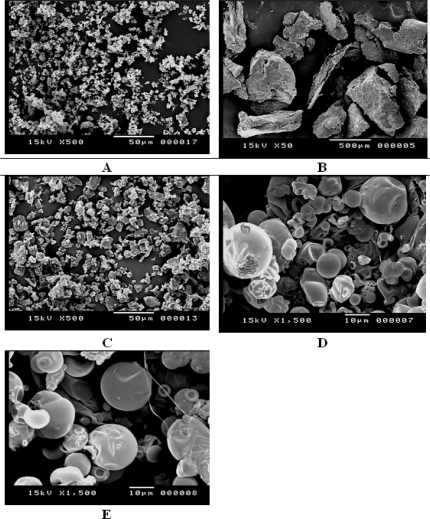
SEM images of meloxicam (A), Gelucire 50/13 (B), physical mixture Gelucire/meloxicam 1:4 (C), solid dispersion Gelucire/meloxicam 1:1 (D) and solid dispersion Gelucire/meloxicam 1:4 (E).

SEM of the raw materials revealed that MX was characterized by irregular shaped crystals whereas Gelucire 50/13 formed big polygonal particles (Fig. 1A, B). The physical mixture at a ratio of 1:4 (MX: Gelucire, w/w; PM) contains the characteristic MX crystals with adhering and free Gelucire 50/13 particles (Fig. 1C). In contrast, the spray dried microparticles prepared from a 1:1 mixture of MX and Gelucire 50/13 represented small homogenous and amorphous aggregates of spherical particles with smooth surfaces (Fig. 1D), while spray dried microparticles prepared from a 1:4 (w/w) mixture of MX and Gelucire were larger spherical particles with rough surfaces (Fig. 1E). Based on the scale, SD particles are smaller than 10 μm.

### Differential scanning calorimetry (DSC)

**Fig. 2.**
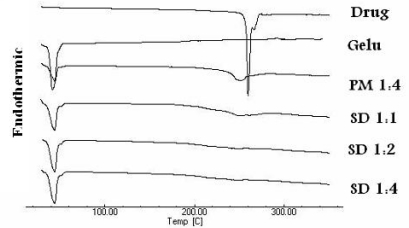
Differential scanning calorimetry of MX (drug), Gelucire (gelu) its physical mixture (PM) and spray dried microparticles (SD) at different ratios of MX/Gelucire (w/w).

Differential scanning calorimetry (DSC) offers information about melting, crystallization, decomposition or a change in heat capacity and is useful to assess the physicochemical status of the entrapped drug as well as the interaction among different compounds. Meloxicam (MX) showed an endothermic peak at 262 °C corresponding to its melting point [16], whereas Gelucire 50/13 showed a broad melting endotherme at 48.5 °C (Fig. 2) [17]. The physical mixture (PM) 1:4 exhibited a weak broad peak at 48.5 °C corresponding to Gelucire 50/13 while the peak corresponding to the drug was shifted to 258 °C and became broad and very weak due to dilution of the drug. In contrast, the characteristic peak of the drug disappeared in all thermograms of spray dried particles. Thus, the crystalline drug was dissolved in the melted carrier and its physical status changed to the amorphous one. This finding is in agreement with Agnivesh et al. 2009 [18], who reported that disappearance of the characteristic peaks of the valsartan dispersed in Gelucire 50/13 is due to melting of the drug in the matrix.

### X-ray diffraction (XRD)

**Fig. 3.**
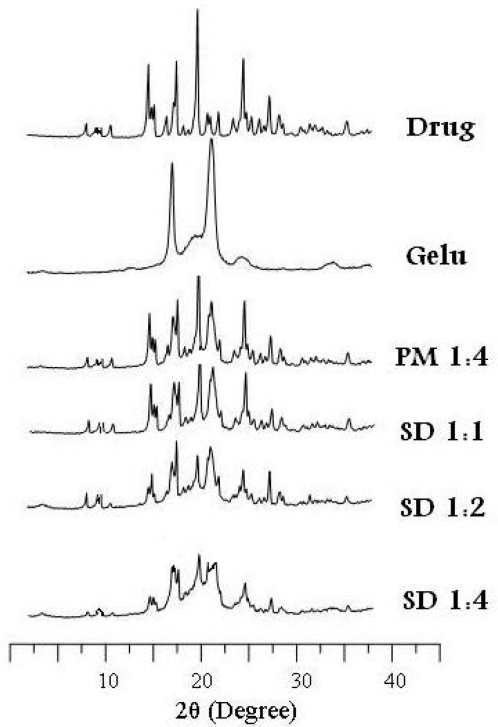
X-ray diffraction of MX (drug), Gelucire (gelu), its physical mixture (PM) and spray dried microparticles (SD) at different ratios of MX/Gelucire (w/w).

As MX is highly crystalline characteristic sharp peaks appeared in XRD at diffraction angles of 2θ at 15.65, 19.31, 19.98, 26.58 and 27.08 (Fig. 3). Gelucire 50/13 also exhibited some crystallinity as indicated by the two characteristic peaks of high intensity at 19.26 and 23.5 at 2θ. All the features already observed for the single compounds were present in their PMs with lower intensity, but no interaction could be detected. The decrease in the intensity of MX-peaks in the diffractograms of the SD-microparticles became apparent at the1:1 ratio and further decreased with decreasing MX-content until complete disappearance at the 1:4 ratio. Thus, the spray drying process decreased the crystallinity or the disordered molecular dispersion of the crystalline drug in the Gelucire matrix during rapid drying of the sprayed droplet. No new peaks could be observed suggesting the absence of any chemical interaction between MX and the matrix [19].

### Solubility study

The solubility of MX was determined in 0.2 M phosphate buffer pH 7.4 at 37 °C (table 1). In general, the presence of Gelucire 50/13 increased the solubility of MX. In case of a PM at the ratio 1:4 (MX:Gelucire, w/w) the solubility of MX was enhanced about 1.64 fold. In contrast, a SD prepared at the same ratio increased the solubility of the drug additionally 3.85 fold. In case of SDs, the increasing solubility of MX with increasing Gelucire-content is higher than that of the PM and might be attributed to the decreasing crystallinity of the drug as confirmed by DSC and XRD.

### Dissolution study

**Fig. 4.**
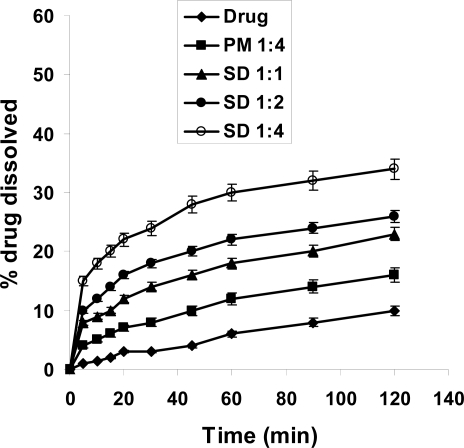
Dissolution profile of MX in 0.1 N HCL from its powder, PM and SDs of MX and Gelucire 50/13.

**Fig. 5.**
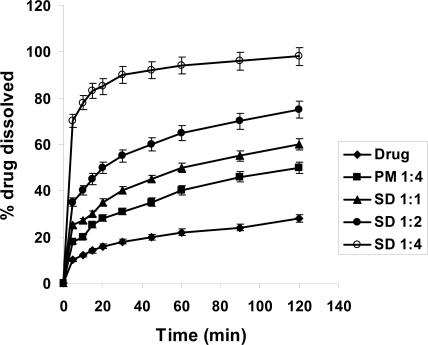
Dissolution profile of MX in 0.2 M phosphate buffer pH 7.4 from its powder, PM and SDs of MX and Gelucire 50/13.

The dissolution rate of MX was extremely low, with only about 10 % and 28 % of drug dissolved after 120 min in 0.1 N HCl and phosphate buffer pH 7.4, respectively (Fig. 4, 5). This might be attributed to poor wettability and particle agglomeration as indicated by floating of the powdered drug on the surface of the media [16]. Similar to that already observed during dissolution studies, the dissolution rate of MX was considerably higher in presence of Gelucire. In artificial gastric juice, the increase in MX-dissolution was about 1.5 fold in case of PM 1:4, while it was about 3.5 fold for SD after 2h. In artificial intestinal juice (Fig. 5) the increase in dissolution of MX was more pronounced. In case of PM the amount of MX dissolved after 2h exceeded that of MX alone 1.5-fold, in case of SD 1:1 2-fold and finally in case of SD 1:4 3.4-fold. This might be due to increased wettability of the drug and decreased surface tension of the medium due to presence of Gelucire [20, 21]. Agnivesh et al. [18] reported that enhancement of dissolution of valsartan from its solid dispersion was due to lack of crystallinity, increased wettability and reduced particle size of the drug. For a more detailed view, the relative dissolution rate (R.D.R.) values were calculated which represent the ratio between the amount of drug dissolved from the formulation (PM or SD) and that of the pure drug (Table 1). Accordingly, the dissolution rate increased following the order SD 1:4 > SD 1:2 > SD 1:1 > PM 1:4. Similar observations have been reported for nifedipine/Pluronic F68 and nifedipine/Gelucire 50/13 solid dispersions where the solubility of poorly water-soluble nifedipine was improved by PM or SDs [17]. Also in case of glibenclamide a similar strategy led to accelerated and improved in-vitro dissolution that enhanced the hypoglycemic effect [22].

### Anti-inflammatory activity

**Fig. 6.**
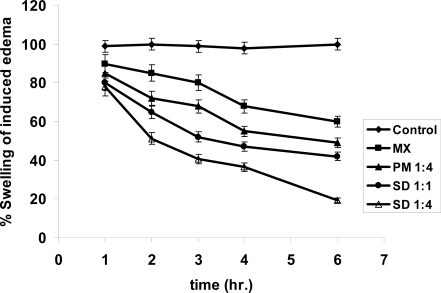
The anti-inflammatory activity of MX and its PM 1:4, SD 1:1 as well as SD 1:4 microparticles.

The anti-inflammatory activity of the formulations was assessed by the carrageenan induced paw edema method scoring the swelling of edema due to presence of the formulations, as compared to the control. Table 2 illustrates the anti-inflammatory activity of different prepared microparticles, PM and drug alone on the hind paw of the rats. All the prepared microparticles exerted significant effects (p < 0.05) as anti-inflammatory vehicles, but at extent (Figure 6). The rank order of edema inhibition applying this method followed the order SD 1:4 > SD 1:1 > PM 1:4 > MX > control.

## Experimental

### Materials

Meloxican (MX) was kindly supplied by Medical Union Pharmaceuticals (MUP) Co. (Abusultan, Ismailia, Egypt). Gelucire 50/13 with m.p. 50 ºC and HLB 13 was provided by Gattefosse (Cedex, France). Aerosil 200 was supplied by ICN Biomedicals, Inc. (OH, USA). All other materials and reagents were of analytical grade.

### Preparation of Physical Mixture

Physical mixtures (PMs) of MX with Gelucire 50/13 at a ratio of 1:4 (w/w) were prepared by blending them for 10 min followed by sieving (250 μm).

### Preparation of solid dispersions (SDs)

A Büchi mini spray dryer model B-191 (Büchi Laboratoriums-Technik AG, Flawil, Switzerland) with standard nozzle (0.7 mm diameter) was used to produce the dry powders of various formulations. MX and Gelucire 50/13 at the ratios of 1:1, 1:2 and 1:4 (w/w) were dissolved in 100 ml ethanol. Silicium dioxide (Aerosil 200) was added to the clear solution at the same amount as that of the drug. The parameters were set as follows: liquid feed 4.5–5.0 ml/min, inlet temperature 70 °C outlet temperature 32°C, and air flow rate 4 psi. The product dried by and stored in a vacuum dessicator at room temperature.

### Scanning Electron Microscopy (SEM)

The samples were sputter coated with gold (SPI, sputter, USA) and images were acquired using a scanning electron microscope (Joel JSM 5400LV SEM, Japan) operated at 15kV.

### Differential Scanning Calorimetry (DSC)

The powdered sample (3–5 mg) was hermetically sealed in aluminum pans and heated from 25°C to 200°C at a constant rate of 10°C/min (DSC-60, Shimadzu, Japan). Data were processed using a TA 50I PC system with Shimadzu software programs. An indium standard was used to calibrate the DSC temperature and enthalpy scale and nitrogen N_2_ as a purging gas at a rate of 40 ml/min.

### X-Ray Powder Diffraction (XRD)

Samples were irradiated with monochromatized Cu Kα radiation (1.542 Å) and analyzed between 2° and 40° (2θ) using a Philips FW 1700 X-ray diffractometer (Philips, Netherlands) setting the voltage at 40 kV and the current at 30 mA.

### Solubility Determination

An excess amount of the sample was dispersed in 0.2 M phosphate buffer pH 7.4. After horizontal shaking of the samples for 48 h at 37 °C, the supernatant was filtered through a Millipore filter (pore size 0.45 μm). 0.5 ml of the filtrate were immediately diluted and assayed spectrophotometrically at 362nm [16]. All experiments were performed in triplicate.

### Release Rate Studies

The USP type II (paddle) method using Electrolab dissolution tester (TDT-06N, India) was adopted. Samples equivalent to 50 mg of pure drug were dispersed in 900 mL of 0.1 N HCl or 0.2 M phosphate buffer (pH 7.4). The dissolution media were maintained at 37°C ± 0.5°C and stirred at 50 rpm. Samples were collected periodically and replaced by fresh dissolution medium. After filtration through a microfilter (0.45 μm), the concentration of MX was determined spectrophotometrically (Jenway 6305 spectrophotometer, UK) at 362 nm. All experiments were carried out in triplicate.

### Anti-inflammatory activity

An acute inflammatory activity model, the carrageenan induced rat paw edema method, was applied in this study [23]. The experimental procedure was in accordance with the guidelines of the Institutional Animal Ethical Committee of King Saud University (approval number KSU/IAEC/1432/12). Rats weighing about 200 ± 20 gm were divided into 5 groups each group has 6 rats. The animals of group 1 received Control (Gelucire 50/13) and group 2, 3,4 and 5 received MX, PM 1:4, SD 1:1 and SD 1:4, respectively. A sample equivalent to 50 mg of each formulation was administered orally to fasted rats with free access to water for 12 hours prior to the test. Inflammation was elicited in the rat’s left hind paw by s.c. injection of 0.1 ml 1.0% w/v carrageenan solution in saline. The rats were anaesthetized with urethane (0.5 ml intraperitoneal). The increase in the paw thickness was measured before carrageenan injection (time 0) and 1, 2, 3, 4 and 6 hrs after carrageenan administration using a dial micrometer. The percentage swelling of the paw and the percent inhibition of edema were calculated according the following equation [24].

$$\%\;\text{swelling}=\frac{V-Vi}{Vi}\times 100$$

Where:

V = the paw thickness at each time interval (mm).
Vi = The initial paw thickness (before carrageenan injection) (mm).

The average paw swelling of treated rats was compared with that of control rats (which received excipients of formulation without drug) and the percent inhibition of edema was determined using the following equation.

$$\%\;\text{inhibition}=\frac{1-\%\text{swelling}\;\text{of}\;\text{treated}\;\text{group}}{\%\text{swelling}\;\text{of}\;\text{control}\;\text{group}}\times 100$$

### Statistical analysis

Statistical analysis for the obtained results was carried out by the student t-test, at 0.05 level of significance.

## Conclusion

Solid dispersions of non steroidal anti-inflammatory drug (MX) were successfully prepared by spray drying technique using Gelucire 50/13. DSC and XRD revealed that the crystallinity of the drug decreased concurrently with increasing amounts of Gelucire. The solubility and dissolution rate of MX was increased concurrent with the Gelucire content of the formulations. Moreover, SDs has superior anti-inflammatory activity than the drug itself and its physical mixture.

## Figures and Tables

**Tab. 1.** Solubility and R.D.R. of MX, PM and SD microparticles in 0.2 M phosphate buffer pH 7.4.

**System**	**Ratio MX:Gelucire (w/w)**	**Solubility (Mx10^−3^)**	**5 min**	**R.D.R. 10 min**	**15 min**
*MX*	–	1.736	–	–	–
*PM*	1:4	2.852	1.80	1.78	1.72
*SD*	1:1	3.752	2.50	2.14	2.22
	1:2	4.850	3.50	3.21	3.05
	1:4	6.685	7.00	5.93	5.00

R.D.R. represents the ratio between the amounts of drug dissolved from formulation and that from drug alone.

**Tab. 2.** The anti-inflammatory activity of MX and its PM 1:4, SD 1:1 as well as SD 1:4.

**Rat group No.**	**System No.**	**% Swelling of induced edema**				
		**1hr**	**2hr**	**3hr**	**4hr**	**6hr**
1	Control	99.0±1.0	100±.05	99.0±0.05	98.01±1.0	100±0.05
2	MX	90.0±1.5 (10.1%)	85.05±1.1 (14.9%)	80.01±0.55 (20.1%)	68.04±0.6 (32.06%)	60.0±1.1 (39.1%)
3	PM 1:4	85.0±0.79 (14.6%)	72.4±0.5 (27.6 %)	68.5±0.3 (32.9%)	55.00±0.6 (44.1%)	49.0±1.5 (51.2%)
4	SD 1:1	80.1±1.5 (19.9%)	65.2±1.2 (34.8%)	52.3±1.8 (48.2%)	47.4±0.9 (53.8%)	42.2±0.9 (58.8%)
5	SD 1:4	78.2±2.0 (22.1%)	51.2±1.5 (48.8%)	40.8±1.2 (58.8%)	30.6±1.4 (68.7%)	19.2±2.1 (80.8%)

The value between parentheses indicates the % inhibition of edema swelling. PM…Physical mixture; SD…Spray drying.
